# Development and Validation of a Novel Nomogram for Preoperative Prediction of In-Hospital Mortality After Coronary Artery Bypass Grafting Surgery in Heart Failure With Reduced Ejection Fraction

**DOI:** 10.3389/fcvm.2021.709190

**Published:** 2021-09-30

**Authors:** Pengyun Yan, Taoshuai Liu, Kui Zhang, Jian Cao, Haiming Dang, Yue Song, Jubing Zheng, Honglei Zhao, Lisong Wu, Dong Liu, Qi Huang, Ran Dong

**Affiliations:** Department of Cardiac Surgery, Beijing Anzhen Hospital, Capital Medical University, Beijing, China

**Keywords:** CABG, HFREF, nomogram, prediction, mortality, EuroSCORE-2

## Abstract

**Background and Aims:** Patients with heart failure with reduced ejection fraction (HFrEF) are among the most challenging patients undergoing coronary artery bypass grafting surgery (CABG). Several surgical risk scores are commonly used to predict the risk in patients undergoing CABG. However, these risk scores do not specifically target HFrEF patients. We aim to develop and validate a new nomogram score to predict the risk of in-hospital mortality among HFrEF patients after CABG.

**Methods:** The study retrospectively enrolled 489 patients who had HFrEF and underwent CABG. The outcome was postoperative in-hospital death. About 70% (*n* = 342) of the patients were randomly constituted a training cohort and the rest (*n* = 147) made a validation cohort. A multivariable logistic regression model was derived from the training cohort and presented as a nomogram to predict postoperative mortality in patients with HFrEF. The model performance was assessed in terms of discrimination and calibration. Besides, we compared the model with EuroSCORE-2 in terms of discrimination and calibration.

**Results:** Postoperative death occurred in 26 (7.6%) out of 342 patients in the training cohort, and in 10 (6.8%) out of 147 patients in the validation cohort. Eight preoperative factors were associated with postoperative death, including age, critical state, recent myocardial infarction, stroke, left ventricular ejection fraction (LVEF) ≤35%, LV dilatation, increased serum creatinine, and combined surgery. The nomogram achieved good discrimination with C-indexes of 0.889 (95%CI, 0.839–0.938) and 0.899 (95%CI, 0.835–0.963) in predicting the risk of mortality after CABG in the training and validation cohorts, respectively, and showed well-fitted calibration curves in the patients whose predicted mortality probabilities were below 40%. Compared with EuroSCORE-2, the nomogram had significantly higher C-indexes in the training cohort (0.889 vs. 0.762, *p* = 0.005) as well as the validation cohort (0.899 vs. 0.816, *p* = 0.039). Besides, the nomogram had better calibration and reclassification than EuroSCORE-2 both in the training and validation cohort. The EuroSCORE-2 underestimated postoperative mortality risk, especially in high-risk patients.

**Conclusions:** The nomogram provides an optimal preoperative estimation of mortality risk after CABG in patients with HFrEF and has the potential to facilitate identifying HFrEF patients at high risk of in-hospital mortality.

## Introduction

The most common cause of heart failure (HF) is coronary artery disease (CAD), which accounts for about 60% of all causes of HF with reduced ejection fraction (HFrEF) (1, 2). For patients with HF, severe left ventricular (LV) systolic dysfunction, and CAD suitable for myocardial revascularization, coronary artery bypass grafting (CABG) is recommended as the first revascularization strategy (3). Despite the recent advances in cardiovascular surgery, CABG among HFrEF patients is still associated with a higher risk of morbidity and mortality than other patients. Therefore, risk assessment is necessary at the time of surgery in patients with HFrEF undergoing CABG. Several risk scores have been developed to help clinicians and patients make informed decisions regarding the risks of surgery. Examples include the Society of Thoracic Surgeons (STS) (4, 5), the EuroSCORE (6), the EuroSCORE-2 (7), and the SinoSCORE risk scores (8). Although helpful, these scores were based on general cardiac surgery patients rather than patients with HFrEF. Additionally, in addition to being outdated and collected more than 10 years ago such scores were developed on western patients, they might be less generalizable to the Chinese patients.

Due to the lack of a specific and practical risk score for HFrEF patients, developing a predictive model that incorporates factors associated with mortality based on preoperative variables is needed. Therefore, this study aims to develop and validate a nomogram score to predict the risk of in-hospital mortality among HFrEF patients with CABG and compare the nomogram score's predictive value with the EuroSCORE-2.

## Materials and Methods

### Study Population

We recruited retrospectively consecutive patients who had undergone CABG in state of HFrEF between January 2013 and July 2019 at Beijing Anzhen Hospital, Capital Medical University. And the HFrEF is commonly defined as a reduction in LVEF to ≤40%, with symptoms and/or signs of heart failure (1, 2). The inclusion criteria included the following: (1) LVEF ≤40% assessed by the last preoperative echocardiography (closest to the time prior to surgery); (2) Symptomatic HF (New York Heart Association [NYHA] functional class II–IV) and; (3) Underwent elective CABG, with or without mitral valve surgery due to ischemic mitral regurgitation. The exclusion criteria included the following: (1) Emergency surgery; (2) Systolic arterial blood pressure <90 mmHg when supine, sitting, or standing; (3) Hemodynamically significant stenotic valvular heart disease; (4) Non-ischemic mitral valve regurgitation caused by papillary muscle rupture, rheumatism, degeneration, infective endocarditis, and congenital heart disease and other organic diseases; (5) Complicated with aortic valve disease, primary myocardiopathy, congenital heart disease, rheumatic heart disease, macrovascular disease or other non-ischemic myocardial diseases; and (6) Cardiogenic shock. Ethical approval was obtained from the Institutional Ethics Committee of Beijing Anzhen Hospital.

### Surgical Procedures

All patients underwent CABG through a midline sternotomy. The left internal mammary artery was the first choice for graft the left anterior descending artery. Saphenous veins and radial arteries were harvested with an open technique, and sequential or separate aortocoronary bypass grafting was performed in the remaining coronary arteries. A transit-time flow probe was used to assess the quality of anastomosis after grafting in all patients. The surgical procedure was jointly decided by more than two experienced surgeons after discussion for patients with mitral regurgitation or ventricular aneurysm. For isolated CABG, the choice of off-pump CABG, on-pump CABG, or On-pump beating heart CABG depended on the surgeon's habit and experience as well as intraoperative conditions.

### Data Collection

Clinical characteristics, echocardiographic findings, laboratory results, and surgical characteristics were collected by trained physicians who are blind to the aim of study with a standard data collection form. In EuroSCORE-2, the critical state is an important variable that included various preoperative conditions and major adverse events. Refer to the definition of critical preoperative state in the EuroSCORE-2, the critical state was defined as a history of ventricular tachycardia or ventricular fibrillation or aborted sudden death, preoperative cardiac massage, preoperative ventilation before anesthetic room, preoperative inotropes, or end-organ damage. Recent myocardial infarction (MI) was defined as MI within 90 days. Increased serum creatinine was defined as serum creatinine measured before surgery >1.5 mg/dl. The echocardiographical parameters, including LVEF and Left ventricular internal diameter at end-diastolic (LVIDd), were extracted from the last preoperative echocardiography (closest to the time prior to surgery). BSA was calculated by Mosteller's formula (9). LVIDd/BSA ≥3.5 cm/m^2^ indicated a moderate or serve Left ventricular (LV) dilatation according to Echocardiography's Guidelines for Chamber Quantification (10). Combined surgery indicated operations combined more than one procedure: include major interventions on the heart such as CABG, mitral valve repair or replacement, and treatment on ventricular aneurysm.

### Clinical Outcome

The primary end point was post-operative mortality during hospitalization. Mortality was defined as any death occurring after a surgical procedure during the hospital stay.

### Statistical Analysis

Continuous variables were expressed as mean ± standard deviation (SD) or median (25th, 75th percentiles) in case of normal or non-normal distribution. The differences between the two groups were examined by independent-sample *t*-test or Mann–Whiney *U*-test, correspondingly. Categorical variables were presented as counts (percentage) and compared by Pearson chi-square test (Pearson χ2 test) or Fisher exact test, as appropriate.

The entire cohort was randomly divided into training cohort and validation cohort (7:3) base on complete data. The training cohort was used to develop the model, and the validation cohort was applied to validate the model. Univariable logistic regression analysis was used to identify the possible predictive factors. The variables with a *p* < 0.15 in univariable analysis and those consistently reported in previous studies were candidates for multivariable logistic regression analysis to identify the independent risk factors for predicting postoperative mortality. We used a backward stepwise elimination approach to simplify the model based on the Akaike Information Criterion. LASSO regression was also applied in the predictors' selection to examine the importance of predictive variables selected by stepwise regression analysis. Based on the selected predictive variables, the logistic regression model was developed and presented as the nomogram.

We assessed the predictive accuracy of the nomogram with discrimination and calibration. To quantify the discrimination performance of the nomogram, Harrell's C-index was measured. The Harrells C statistic is a measure of discrimination that is similar to the area under a receiver operating characteristic curve (ROC) (11). Calibration curves were plotted to assess the calibration of the nomogram, accompanied with the Hosmer-Lemeshow test [A significant test statistic implies that the model doesn't calibrate perfectly (12)]. To further assess model calibration, predicted probabilities for mortality were calculated for participants in the training cohort, divided into quintiles, and compared with observed mortality. The results were presented as a bar chart. To decrease the overfit bias and increase precision, the nomogram model was subjected to bootstrapping validation (1000 bootstrap resamples) to evaluate a relatively corrected C-index and calibration ability in the training cohort. To assess the performance of the nomogram in the validation cohort, the logistic regression formula developed in the training cohort was then applied in the validation cohort, with predicted postoperative mortality calculated. Finally, the C-index, the calibration curve, and the Hosmer-Lemeshow test were used.

EuroSCORE-2 online calculator (http://www.euroscore.org) was used to calculate the predicted mortality of each patient. DeLong's test was used to compare C-index between the nomogram and the EuroSCORE-2 in the training and validation cohort, respectively. Besides, we calculated the categorical net reclassification improvement (NRI) and integrated discrimination improvement (IDI) to determine the extent to which the predictive power of the nomogram is better than EuroSCORE-2. Calibration of the two models was evaluated and compared by the Hosmer-Lemeshow statistic χ2 and *P* > 0.05 indicates the model fits well. Similarly, the two models were visualized graphically by comparing the observed probability with the predicted probability of death across quintiles of predicted risk.

The present study is reported in compliance with standard guidelines (13) for prediction models and the transparent reporting of a multivariable prediction model for individual prognosis or diagnosis (TRIPOD) checklist is presented in Supplementary Material. Statistical analysis was conducted in R software (version 4.0.2; http://www.Rproject.org). C-index, calibration curve, nomogram, and bootstrapping validation were calculated or formulated using rms and riskRegression packages in R. NRI and IDI were calculated with PredictABEL packages in R. A two-tailed *p* < 0.05 indicated statistical significance. Data analysis was conducted from November 7, 2020 to February 24, 2021.

## Results

A total of 489 consecutive patients were enrolled in the present study. Of these, 36 patients (7.4%) died after surgery. The perioperative complications included prolonged ventilation >96 hours in 36 (7.36%) patients, reoperation for any reason in 23 (4.70%), reoperation for bleeding in 9 (1.84%), tracheostomy in 11 (2.25%), renal failure requiring continuous renal replacement therapy (CRRT) in 24 (4.91%), stroke in 12 (2.45%), cardiac arrest requiring cardiopulmonary resuscitation (CPR) in 14 (2.86%), and implantation of extracorporeal membrane oxygenation (ECMO) in 11 (2.25%).

We randomly allocated 70% (342) of patients to the training cohort and the remainder 30% (147) to a validation cohort. There were 26(7.6%) and 10(6.8%) patients who died after surgery in the training and validation cohorts, respectively. The baseline characteristics in all cohorts are listed in Table 1. There were no significant differences between the training and validation cohorts regarding preoperative baseline and surgical characteristics.

**Table 1:** Baseline characteristics of patients.

**Variable**	**Entire cohort (*n* = 489)**	**Training cohort (*n* = 342)**	**Validation cohort (*n* = 147)**	***P*-Value**
Age (years)	60.0 (52.0,67.0)	60.0 (52.2,66.0)	60.0 (52.0,67.0)	0.746
Female	75 (15.3%)	53 (15.5%)	22 (15.0%)	0.990
BMI (kg/m^2^)	25.2 (23.4,27.3)	25.4 (23.5,27.4)	24.9 (23.3,27.0)	0.359
Critical state	13 (2.7%)	9 (2.6%)	4 (2.7%)	1.000
Hypertension	243 (49.7%)	169 (49.4%)	74 (50.3%)	0.929
Diabetes mellitus	233 (47.6%)	163 (47.7%)	70 (47.6%)	1.000
Diabetes on insulin	82 (16.8%)	50 (14.6%)	32 (21.8%)	0.071
Hyperlipidemia	151 (30.9%)	107 (31.3%)	44 (29.9%)	0.849
Smoke	280 (57.3%)	200 (58.5%)	80 (54.4%)	0.464
Alcohol	105 (21.5%)	67 (19.6%)	38 (25.9%)	0.154
Chronic kidney disease	8 (1.6%)	6 (1.8%)	2 (1.4%)	1.000
Dialysis	4 (0.8%)	2 (0.6%)	2 (1.4%)	0.587
Chronic pulmoriary disease	19 (3.9%)	16 (4.7%)	3 (2.0%)	0.259
Stroke	74 (15.1%)	51 (14.9%)	23 (15.6%)	0.944
PCI	116 (23.7%)	84 (24.6%)	32 (21.8%)	0.582
Previous cardiac surgery	2 (0.4%)	1 (0.3%)	1 (0.7%)	0.511
Pulmonary hypertension	16 (3.3%)	12 (3.5%)	4 (2.7%)	0.786
Recent MI	126 (25.8%)	87 (25.4%)	39 (26.5%)	0.888
NYHA class III/IV	77 (15.7%)	52 (15.2%)	25 (17.0%)	0.714
CCS angina class = 4	19 (3.9%)	15 (4.4%)	4 (2.7%)	0.536
Carotid artery stenosis	150 (30.7%)	101 (29.5%)	49 (33.3%)	0.466
Lower limb arterial stenosis	174 (35.6%)	120 (35.1%)	54 (36.7%)	0.806
LVEF ≤ 35%	161 (32.9%)	109 (31.9%)	52 (35.4%)	0.515
LV dilatation	110 (22.5%)	68 (19.9%)	42 (28.6%)	0.046
Ischemic mitral regurgitation				0.151
No	253 (51.7%)	188 (55.0%)	65 (44.2%)	
Mild	111 (22.7%)	75 (21.9%)	36 (24.5%)	
Moderate	87 (17.8%)	55 (16.1%)	32 (21.8%)	
Severe	38 (7.8%)	24 (7.0%)	14 (9.5%)	
Ventricular aneurysm	135 (27.6%)	95 (27.8%)	40 (27.2%)	0.985
Increased serum creatinine	20 (4.1%)	14 (4.1%)	6 (4.1%)	1.000
CPB				0.064
OP	323 (66.1%)	236 (69.0%)	87 (59.2%)	
ONBEAT	72 (14.7%)	49 (14.3%)	23 (15.6%)	
ONSTOP	94 (19.2%)	57 (16.7%)	37 (25.2%)	
MVP	36 (7.4%)	25 (7.3%)	11 (7.5%)	1.000
MVR	18 (3.7%)	10 (2.9%)	8 (5.44%)	0.274
Intervention on ventricular aneurysm	60 (12.3%)	38 (11.1%)	22 (15.0%)	0.298
Combined surgery	104 (21.3%)	68 (19.9%)	36 (24.5%)	0.307

*BMI, Body mass index; MI, myocardial infarction; PCI, percutaneous coronary intervention; NYHA, New York heart association; CCS, Canadian cardiovascular society; LVEF, left ventricular systolic function; CPB, Cardiopulmonary bypass; CABG, coronary artery bypass grafting; OP, Off-pump CABG without CPB; ONBEAT, On-pump beating heart CABG; ONSTOP, On-pump CABG; MVR, mitral valve replacement; MVP, mitral valve replacement*.

### Univariable Analysis

The results of the univariable logistic regression analysis of predictors associated with postoperative mortality in the training cohort are presented in Table 2. Univariable analysis in the training cohort showed a significant association of postoperative morality with several predictors including age, critical state, diabetes on insulin, stroke, recent myocardial infarction (MI) within 90 days, CCS angina class IV, lower limb arterial stenosis, left ventricular ejection fraction (LVEF) ≤35%, LV (left ventricular) dilatation, ischemic mitral regurgitation, increased serum creatinine, and mitral valve replacement (MVR).

**Table 2:** Univariable Logistic Regression Analysis of predictors associated with in-hospital mortality in the Training Cohort.

**Variables**	**OR (95%CI)**	***P*-value**
Age (years)	1.07 (1.02–1.12)	0.007
Sex, male vs. female	2.18 (0.80–5.33)	0.119
BMI (kg/*m*^2^)	0.89 (0.78–1.02)	0.084
Critical State, yes vs. no	11.2 (2.50–47.0)	0.003
Hypertension, yes vs. no	0.74 (0.32–1.66)	0.461
Diabetes mellitus, yes vs. no	1.83 (0.81–4.32)	0.148
Diabetes on insulin, yes vs. no	3.56 (1.42–8.42)	0.008
Hyperlipidemia, yes vs. no	0.65 (0.23–1.59)	0.361
Smoke, yes vs. no	0.69 (0.31–1.56)	0.371
Alcohol, yes vs. no	0.75 (0.21–2.07)	0.608
Chronic renal insufficiency, yes vs. no	6.64 (0.79–38.1)	0.076
Dialysis, yes vs. no	12.4 (0.31–490)	0.152
Chronic pulmoriary disease, yes vs. no	1.90 (0.26–7.47)	0.460
Stroke, yes vs. no	2.84 (1.09–6.80)	0.033
PCI, yes vs. no	1.71 (0.70–3.95)	0.232
Previous cardiac surgery, yes vs. no	–	–
Pulmonary hypertension, yes vs. no	1.25 (0.05–6.91)	0.848
Recent MI, yes vs. no	3.86 (1.69–8.92)	0.001
NYHA class, III/IV vs. I/II	2.00 (0.85–4.53)	0.108
CCS angina class, IV vs. I/II/III	5.10 (1.28–16.6)	0.023
Carotid artery stenosis, yes vs. no	0.71 (0.25–1.74)	0.472
Lower limb arterial stenosis, yes vs. no	2.72 (1.21–6.33)	0.016
LVEF, ≤35 vs. >35%	3.80 (1.67–9.05)	0.001
**LV dilatation**		
LVIDd/BSA <3.5 cm/m^2^	Reference	Reference
LVIDd/BSA ≥3.5 cm/m^2^	8.01 (3.46–19.4)	<0.001
**Ischemic mitral regurgitation**		
No	Reference	Reference
Mild	2.31 (0.77–6.81)	0.132
Moderate	4.36 (1.56–12.4)	0.005
Severe	2.14 (0.28–9.49)	0.405
Ventricular aneurysm, yes vs. no	0.78 (0.27–1.91)	0.602
Increased serum creatinine, yes vs. no	8.11 (2.26–26.3)	0.002
**CPB**		
OP	Reference	Reference
ONBEAT	2.23 (0.74–5.98)	0.144
ONSTOP	1.89 (0.63–5.01)	0.240
MVP, yes vs. no	1.13 (0.16–4.19)	0.879
MVR, yes vs. no	5.85 (1.13–23.3)	0.037
**Intervention on ventricular aneurysm**		
No	Reference	Reference
Yes	2.08 (0.65–5.56)	0.201
Combined surgery, yes vs. no	2.32 (0.94–5.39)	0.068

*OR, odds ratio; CI, confidence interval; BMI, Body mass index; MI, myocardial infarction; PCI, percutaneous coronary intervention; NYHA, New York heart association; CCS, Canadian cardiovascular society; LVEF, left ventricular systolic function; LVIDd, left ventricular internal diameter at end-diastolic; BSA, Body Surface Area; CPB, Cardiopulmonary bypass; MVR, mitral valve replacement; MVP, mitral valve replacement; CABG, coronary artery bypass grafting; OP, Off-pump CABG without CPB; ONBEAT, On-pump beating heart CABG; ONSTOP, On-pump CABG*.

### Multivariable Analysis

**Figure 1:**
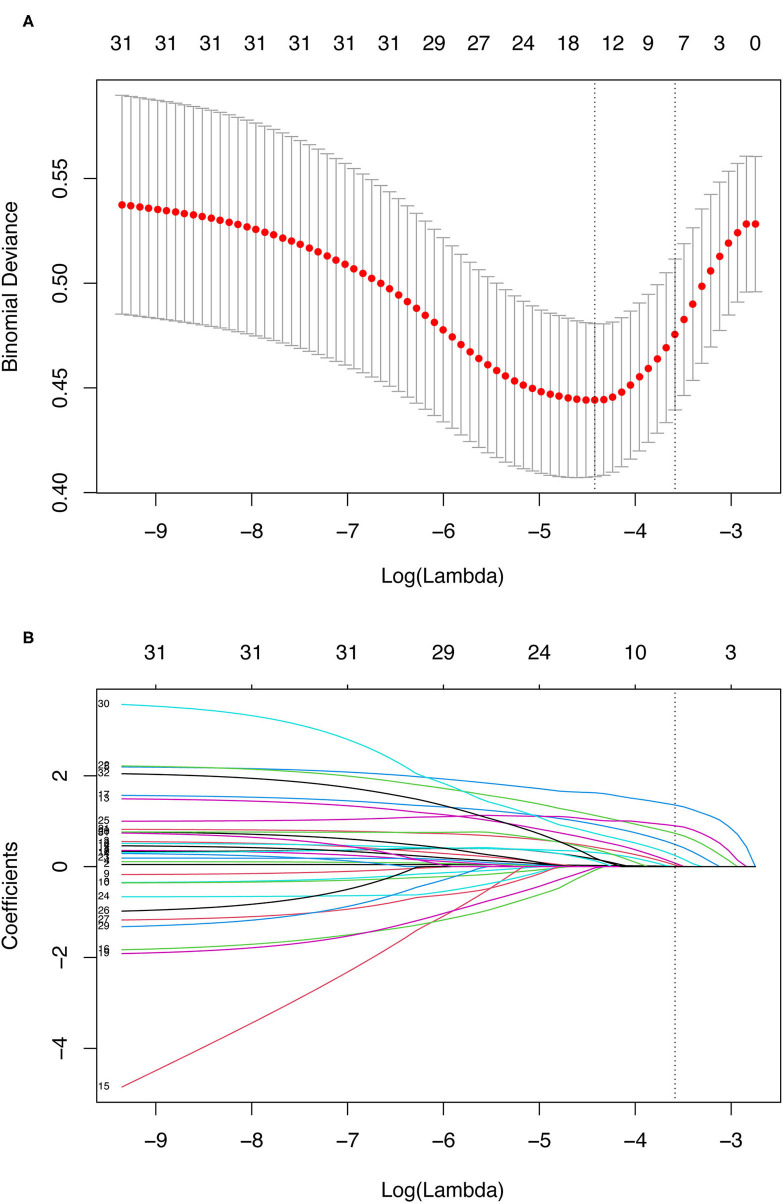
Demographic and clinical variables selection using the LASSO binary logistic regression model. **(A)** Optimal parameter (lambda) selection in the LASSO model used tenfold cross-validation *via* minimum criteria. The binomial deviance curve was plotted vs. log(lambda). Dotted vertical lines were drawn at the optimal values by using the minimum criteria and the 1 SE of the minimum criteria (the 1-SE criteria). **(B)** LASSO coefficient profiles of the 31 variables. A coefficient profile plot was produced against the log(lambda) sequence. A dotted vertical line was drawn at the value selected using tenfold cross-validation, where optimal lambda (with the 1-SE criteria) resulted in eight predictive variables the same as variables selected by the stepwise regression method. LASSO, least absolute shrinkage and selection operator; SE, standard error.

Multivariable logistic regression analysis demonstrated that age, critical state, recent MI within 90 days, stroke, LVEF ≤35%, left ventricular (LV) dilatation, increased serum creatinine, and combined surgery remained significant independent risk factors for postoperative mortality. The β-coefficients, odds ratios, 95% confidence interval (CI) and *p*-values for each of the variables in the multivariable analysis are displayed in Table 3. What's more, LASSO regression also resulted in eight predictive variables the same as the variables selected by the stepwise regression method (Figure 1).

**Table 3:** Multivariable logistic regression analysis of predictors associated with in-hospital mortality in the Training Cohort.

**Variable**	**β coefficient**	**OR (95% CI)**	***P*-value**
Age	0.058	1.06(1.01–1.11)	0.016
Critical State	1.990	7.31(1.68–30.15)	0.006
Recent MI	1.601	4.96(2.12–12.09)	<0.001
Stroke	1.017	2.77(1.08–6.85)	0.029
LVEF≤35%	0.887	2.43(1.06–5.65)	0.036
LV dilatation	1.579	4.85(2.06–11.82)	<0.001
Increased serum creatinine	1.423	4.15(1.00–16.19)	0.043
Combined surgery	1.185	3.27(1.42–7.49)	0.005
(Intercept)	−8.538		<0.001

*OR, odds ratio; CI, confidence interval; MI, myocardial infarction; LVEF, left ventricular ejection fraction*.

### Nomogram Derived From the Training Cohort

**Figure 2:**
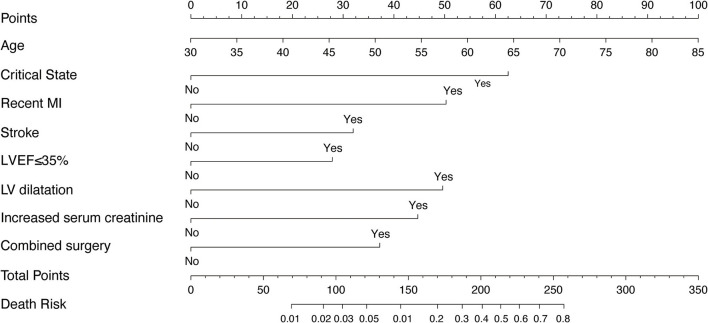
The nomogram derived from training cohort for predicting mortality after CABG. MI, myocardial infarction; LVEF, left ventricular ejection fraction.

**Figure 3:**
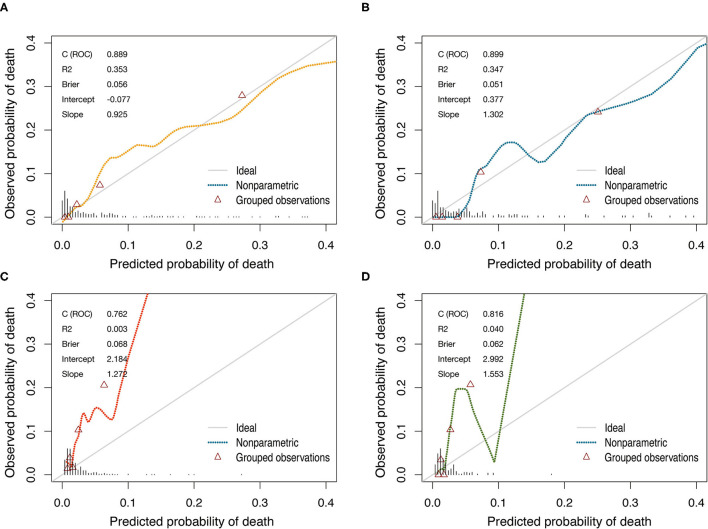
Calibration curves of the nomogram and the EuroSCORE-2 for predicting mortality after CABG in training cohort and validation cohort. **(A)** The nomogram in training cohort. **(B)** The nomogram in validation cohort. **(C)** The EuroSCORE-2 in training cohort. **(D)** The EuroSCORE-2 in validation cohort. Diagonal line indicates perfect calibration. The triangles indicate the observed frequencies of death by the quintiles of the predicted probability.

The model that integrated selected predictive factors was developed and presented as the nomogram (Figure 2). The C-index for death risk prediction in the training cohort was 0.889 (95%CI, 0.839–0.938; Table 4), which was confirmed to be 0.823 (the corrected C-index) *via* bootstrapping validation. The Hosmer-Lemeshow test yielded a non-significant statistic (*p* = 0.535), which suggested no departure from a perfect fit. For the patients whose predicted mortality probabilities were below 40%, the calibration curve demonstrated an optimal agreement between the prediction by nomogram and actual observation (Figure 3A). In addition, the calibration curve with bootstrap similarly showed good calibration in patients in whom the predicted mortality probabilities were below 40% (Supplementary Figure 1).

**Table 4:** Comparison of the nomogram and the EuroSCORE-2.

	**Training cohort (*****n*** **=** **342)**			**Validation cohort (*****n*** **=** **147)**		
	**EuroSCORE-2**	**Nomogram**	***P*-value**	**EuroSCORE-2**	**Nomogram**	***P*-value**
C-index (95% CI)	0.762 (0.661–0.863)	0.889 (0.839–0.938)	0.005	0.816(0.705–0.928)	0.899(0.835–0.963)	0.039
Categorical NRI (95% CI)	Reference	0.471 (0.287–0.655)	<0.001	Reference	0.572 (0.367–0.776)	<0.001
IDI (95% CI)	Reference	0.202 (0.112–0.291)	<0.001	Reference	0.157 (0.072–0.243)	<0.001
χ-squared (Hosmer-Lemeshow test)	77.337	7.016	–	24.998	5.694	–
*P*-value (Hosmer-Lemeshow test)	<0.001	0.535	–	0.002	0.682	–

*NRI, net reclassification improvement, IDI, integrated discrimination improvement, CI, confidence interval*.

### Validation of Predictive Accuracy of the Nomogram in the Validation Cohort

**Figure 4:**
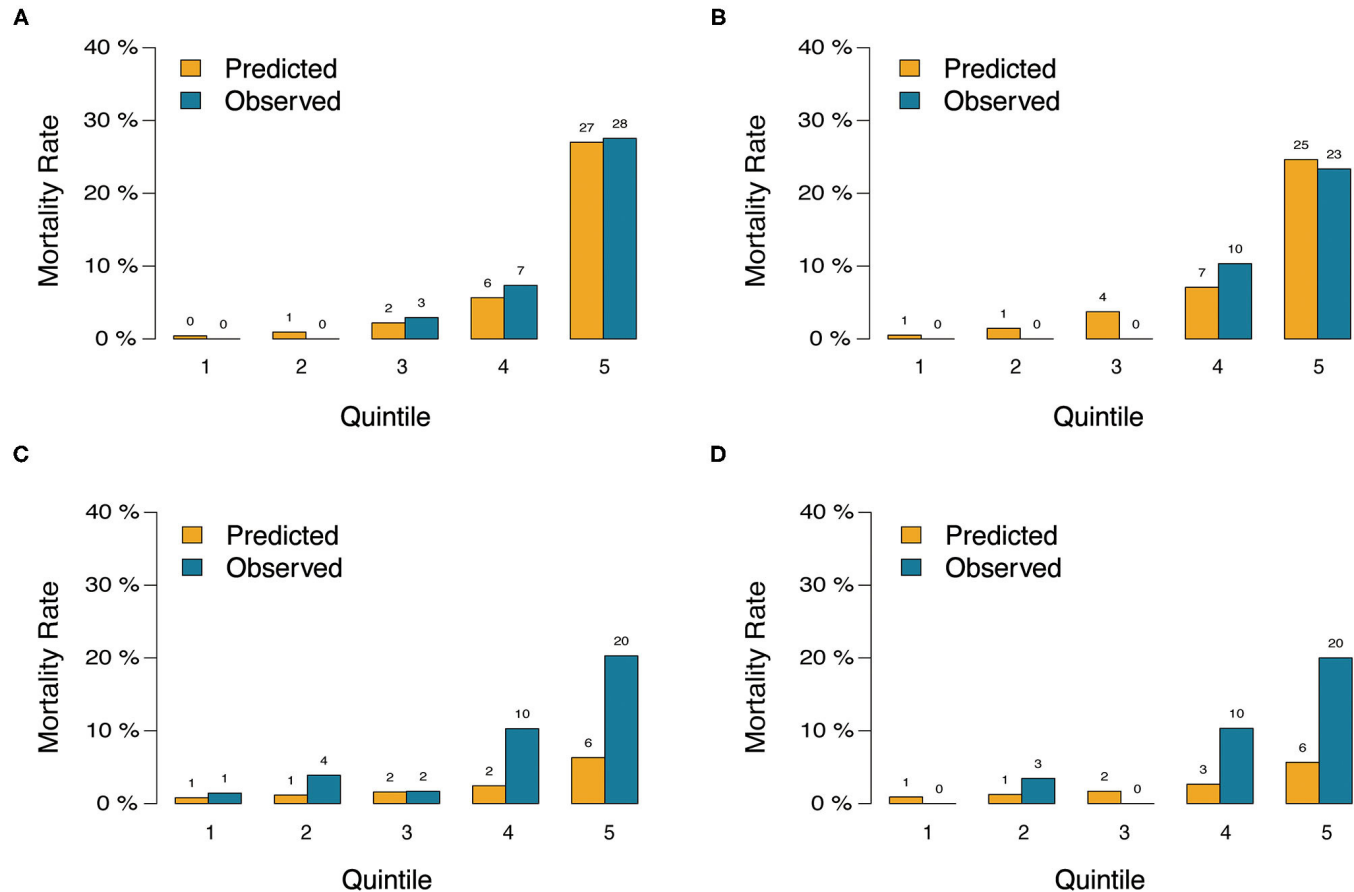
Predicted vs. observed risk of death after CABG based on quintile of predicted risk in training cohort and validation cohort. **(A)** the nomogram in training cohort. **(B)** The nomogram in validation cohort. **(C)** The EuroSCORE-2 in training cohort. **(D)** The EuroSCORE-2 in validation cohort.

In the validation cohort, the C-index of the nomogram for predicting postoperative mortality was 0.899(95%CI, 0.835–0.963; Table 4). There was no significant difference regarding the C-index between the training and validation cohort (0.889 vs. 0.899, *p* = 0.804). The Hosmer-Lemeshow test similarly yielded a non-significant statistic (*p* = 0.682) indicating acceptable goodness-of-fit. For patients with predicted mortality probabilities below 40%, the calibration curve also showed accepted agreement between prediction and observation in the probability of mortality (Figure 3B). Model calibration of the nomogram was further explored by comparing the predicted and observed probabilities across predicted risk quintiles. It showed that the nomogram had an acceptable agreement between prediction and observation both in the training and validation cohort (Figures 4A,B). The nomogram derived from the training cohort displayed good discrimination and calibration in predicting postoperative mortality both in the training and validation cohort.

### Comparison of Predictive Accuracy Between the Nomogram and EuroSCORE-2

**Figure 5:**
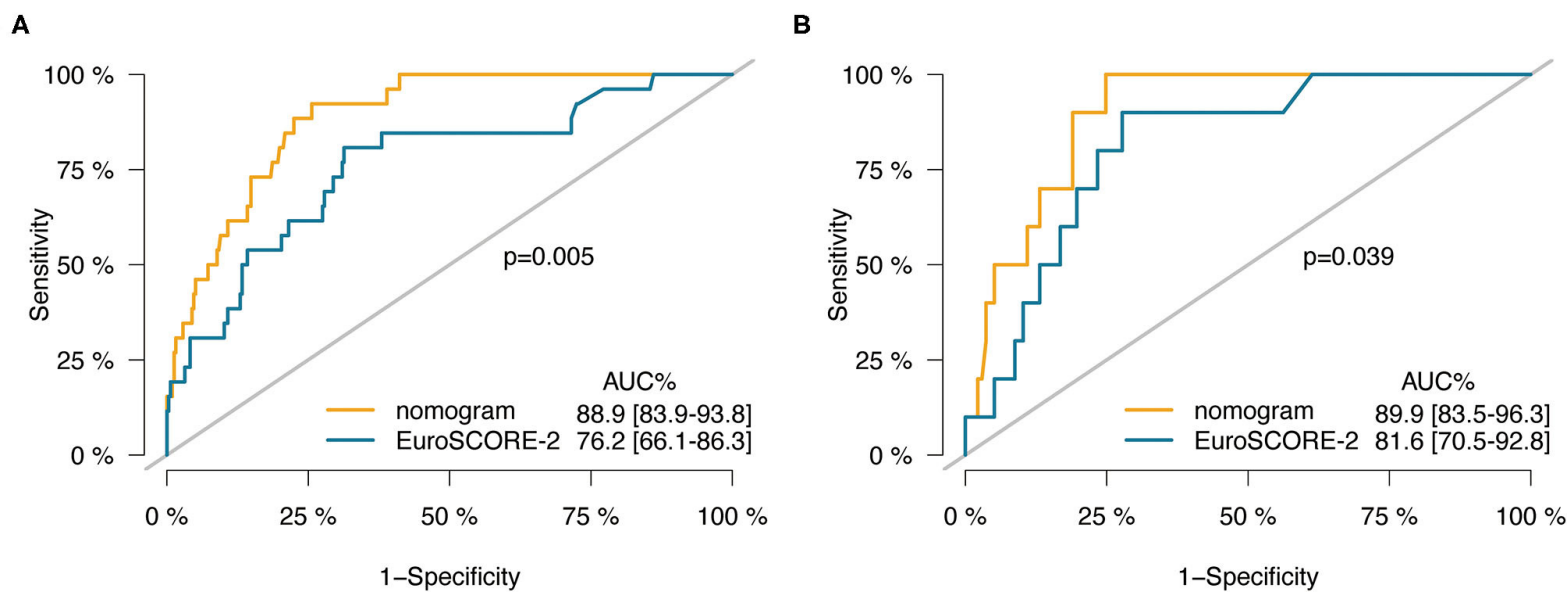
ROC curves of the nomogram vs. the EuroSCORE-2 in training cohort **(A)** and validation cohort **(B)**.

The C-index of the nomogram was significantly higher than the EuroSCORE-2 in training (0.889 vs. 0.762, *p* = 0.005) and validation cohort (0.899 vs. 0.816, *p* = 0.039; Table 4 and Figure 5). The reclassification and discrimination ability of the nomogram was assessed. Compared with EuroSCORE-2, the nomogram showed significantly improved prediction performance in training cohort (categorical NRI: 0.471 [0.287–0.655], *p* < 0.001; IDI: 0.202 [0.112–0.291], *p* < 0.001) and validation cohort (categorical NRI: 0.572 [0.367–0.776], *p* < 0.001; IDI: 0.157 [0.072–0.243], *p* < 0.001; Table 4).

The nomogram had acceptable calibration in training (Hosmer-Lemeshow statistic χ^2^ = 7.016, *p* = 0.535) and validation cohort (Hosmer-Lemeshow statistic χ^2^ = 5.694, *p* = 0.682; Table 4). For EuroSCORE-2, the Hosmer-Lemeshow test yielded a significant statistic in training (Hosmer-Lemeshow statistic χ^2^ = 77.337, *p* < 0.001) and validation cohort (Hosmer-Lemeshow statistic χ^2^ = 24.998, *p* = 0.002), indicating that the EuroSCORE-2 does not calibrate perfectly. For patients with an expected mortality rate of <40%, the calibration curve of the nomogram indicated a good fit of predicted and observed mortality in the training and validation cohort (Figures 3A,B). As for EuroSCORE-2, the calibration curve showed poor agreement between prediction and observation in the probability of mortality in the training and validation cohort. The calibration curve was almost above the 45° diagonal line, which means EuroSCORE-2 underestimated the probability of mortality, especially in high-risk patients (Figures 3C,D). Model calibration was further explored by comparing the predicted and observed probabilities of mortality across patient predicted risk quintiles. It also shows that EuroSCORE-2 underestimated the probability of mortality in high-risk patients (Figures 4C,D).

## Discussion

To our knowledge, we developed the first nomogram model to efficiently predict the in-hospital mortality after CABG among patients with HFrEF. The nomogram risk prediction model performed well in our training and validation cohorts, and showed good discrimination and calibration in patients with predicted mortality probabilities below 40%. The model incorporates only eight preoperative variables which are easily measured and readily available: age, critical state, recent MI, stroke, LVEF ≤35%, left ventricular dilatation, increased serum creatinine, and combined surgery.

### Risk Factors

The nomogram incorporates only 8 variables but achieved good model performance. We can conclude that the 8 risk factors included in the nomogram are the most important variables associate with mortality in patients with HFrEF undergoing CABG. It is well-established that age independently affects post-CABG mortality, and was included in the nomogram. Contrary to commonly used risk scores, sex and BMI were not independent risk factors in the nomogram. In EuroSCORE-2, previous cardiac surgery and critical state were two risk factors given the heaviest weight. Similarly, the critical state was given the heaviest weight in the nomogram. However, previous cardiac surgery wasn't included in our model because only two patients had a history of cardiac surgery in the entire cohort and accounted for a very small proportion in our cohort. A growing number of literatures documented the effects of renal dysfunction on mortality and morbidity after CABG surgery (14–18). Serum creatinine is often used to reflect renal function because it is readily available and simple. It was reported that patients with a baseline serum creatinine of more than 1.5 mg/dl had a significantly higher 30-day mortality after CABG (15). Consistent with those reports, in our model, we defined increased serum creatinine as serum creatinine >1.5 mg/dl and similarly found it was independently associated with increased postoperative mortality in patients with HFrEF. The combined surgery not only reflects more severe lesions that need additional intervention of mitral valve or ventricular aneurysm, but also reflects a longer time of anesthesia and use of cardiopulmonary bypass (CPB). These factors increased the risk of surgery but also might have encouraged surgeons to change or simplify operative procedures to limit anesthesia time and avoid cardiopulmonary bypass.

One of the most powerful predictors of in-hospital mortality in our study was LV dilatation. Yamaguchi et al. (19) revealed that preoperative LV end-systolic volume index (LVESVI) >100 ml/m^2^ predicted the development of congestive HF and late mortality in patients with LVEF <30% undergoing isolated CABG. The results from Surgical Treatment for Ischemic Heart Failure (STICH) Trial (20) showed that, in patients with left ventricular dysfunction who underwent CABG, LVESVI was a stronger predictor of 30-day mortality than LVEF, and mortality risk increased linearly with increasing values of LVESVI. Fukunaga et al. (21) found that LV size >5.5 cm was a significant predictor of operative mortality and major morbidity (OR 5.5 [2.0–15.7] (*p* < 0.001) in patients undergoing isolated CABG. Our study defined LVIDd/BSA ≥3.5 cm/m^2^ as moderate or serve LV dilatation. Similarly, we found LV dilatation was a significant risk factor of in-hospital mortality and showed stronger predictive ability than LVEF. Well-accepted surgical risk scores have identified only LVEF as a powerful predictor of surgical and 30-day mortality, which may be inaccurate. A variable reflecting LV size may be a more important predictor of outcome than LVEF and should be incorporated into their risk-adjustment models.

### The Advantages of Nomogram Compared With the EuroSCORE-2

EuroSCORE-2 and STS score are the most commonly used risk scores and have been proven effective in assessing postoperative risk for general patients undergoing cardiac surgery (22–24). However, these scores were based on data including only a small number of patients with HFrEF and may not be accurate to predict surgery risk in such high-risk patients. Howell et al. (25) showed that EuroSCORE-2 performed not well with a low C-statistic of 0.67 and poor model calibration (chi-square 16.5; *p* = 0.035) in high-risk patients who underwent cardiac surgery (preoperative logistic EuroSCORE ≥10). Several pieces of literature reported that EuroSCORE-2 or STS score had underestimated surgery mortality of CABG when applied to specific high-risk populations (26–28). Di Dedda et al. revealed that, EuroSCORE-2 significantly underestimated the mortality risk (predicted mortality 6.5%) in high-risk patients with cardiac surgery (observed mortality 11%). (26). In patients with an LVEF ≤35% undergoing CABG, it has been reported (29) that both the STS Score and the EuroSCORE-2 performed moderately well, but with a C-index (C statistic is <0.75), somewhat inferior to that reported for overall cardiac surgical populations (where their C statistic is >0.80). What's more, both the STS score and EuroSCORE-2 significantly underestimated mortality. The STS score appeared to consistently underestimate risk compared with the EuroSCORE-2. Consistent with these reports, in our study, EuroSCORE-2 had a moderate C-index (0.762 and 0.816 in training and validation cohort) and similarly significantly underestimated the risk of mortality after CABG in patients with HFrEF as shown in the calibration curve, especially in the high-risk group.

Unlike Western countries, China is a developing country and has different medical standards and characteristics. For example, Off-pump CABG is more common than on-pump CABG in china. Thus, based on populations in Europe and the US, EuroSCORE-2 and STS score are not suitable for Chinese patients. Moreover, the data of EuroSCORE-2 and STS score were obtained from more than 10 years ago, which could be outdated with the improvements in surgical, anesthetic and intensive care during the past decade. Consequently, a new model developed for specific Chinese patients with HFrEF undergoing CABG is urgently needed.

In this study, we established a nomogram prediction model that showed favorable discrimination with C-index consistently more than 0.8 and significantly higher than EuroSCORE-2 in the training and validation cohort. Besides, the nomogram showed a better calibration than EuroSCORE-2 in both cohorts. We thought it might be attributed to reasons as followed: First, our nomogram was specifically developed for patients with HFrEF instead of general patients. Second, the nomogram was developed using data from the last 8 years. However, EuroSCORE-2 was based on data obtained from more than 10 years ago, which could be outdated. Third, it has been reported that EuroSCORE-2 underestimated mortality in the high-risk Chinese patients undergoing CABG (27). Different from EuroSCORE-2 that based on the western population, our nomogram is more suitable for Chinese patients. Fourth, LV dilatation is a more important predictor of outcome than LVEF and was incorporated in the nomogram but not in the EuroSCOR-2. Finally, our risk model developed from single-center data with internal validation instead of external validation. The performance of the nomogram in external validation may not be that good.

Furthermore, our nomogram has unique advantages over traditional risk scores. It has only eight risk factors generally included in the medical records and was easier to calculate risk bedside in a few minutes and worthy of clinical popularizing. However, the STS score is complex, with more than 50 demographic and operative variables, and even EuroSCORE-2 has 18 variables. Despite fewer variables for prediction, our nomogram had demonstrated better predictive performance in calibration and discrimination than EuroSCORE-2. With fewer variables but achieved better model performance, this study demonstrates the utility and feasibility of using specific patient data for constructing models to improve prediction of cardiac surgery mortality in specific populations and gain additional insight into factors that modify the risk of outcomes in patients with HFrEF.

### Limitation

There are several limitations in this study. First, this study is a retrospective analysis, and hence selection bias remains a possibility and prospective studies are required to confirm the results. Second, our risk model was developed from single-center data without external validation. Although we tried to overcome this limitation by internal validation in the validation cohort and additional validation with the bootstrap method, external validation in other cohorts is needed before clinical application. Third, the nomogram model was developed and validated in a small cohort with only 36 outcomes. Considering the relatively small sample size, results from this study should be interpreted with caution. The present study is a preliminary explore in predicting risk of mortality in these specific high-risk patients with CABG. And future studies with large sample size are needed to further confirm our findings. Finally, the model was based on routine clinical data, some potentially important predictor variables were not collected, such as natriuretic peptide levels. Specific markers to estimate surgery risk might further improve the accuracy of the model.

## Conclusion

In conclusion, this study presents an easily applied nomogram that can predict in-hospital mortality in HFrEF patients undergoing CABG. This nomogram showed an improvement in the predictive accuracy when compared to EuroSCORE-2. The nomogram may help identify HFrEF patients at high risk of in-hospital mortality after CABG who might benefit from a simplified operation approach, perioperative intense attention, and more personalized treatment.

## Data Availability Statement

The raw data supporting the conclusions of this article will be made available by the authors, without undue reservation.

## Ethics Statement

The studies involving human participants were reviewed and approved by Ethical approval was obtained from the Institutional Ethics Committee of Beijing Anzhen Hospital. The patients/participants provided their written informed consent to participate in this study.

## Author Contributions

PY, RD, TL, KZ, and JC: study concept and design. All authors acquisition, analysis, or interpretation of data, critical revision of the manuscript for important intellectual content, and read and approved the final manuscript. PY: drafting of the manuscript.

## Funding

This study was supported by a grant from the National Natural Science Foundation of China (81770412), Beijing Municipal Science and Technology Commission (Z151100003915084), and Beijing Municipal Health Commission Capital Health Development Scientific Research Project (shoufa 2020-2Z-2067).

## Conflict of Interest

The authors declare that the research was conducted in the absence of any commercial or financial relationships that could be construed as a potential conflict of interest.

## Publisher's Note

All claims expressed in this article are solely those of the authors and do not necessarily represent those of their affiliated organizations, or those of the publisher, the editors and the reviewers. Any product that may be evaluated in this article, or claim that may be made by its manufacturer, is not guaranteed or endorsed by the publisher.
